# Intra-Articular Osteoid Osteoma as a Cause of Anteromedial Knee Pain

**DOI:** 10.1155/2017/5846368

**Published:** 2017-05-02

**Authors:** Ergin Sagtas, Kemal Gokkus, Ahmet Turan Aydin

**Affiliations:** ^1^Department of Radiology, Memorial Antalya Hospital, Zafer Mah. Yildirim Beyazit Cad. No. 91, Kepez, Antalya, Turkey; ^2^Department of Orthopedic Surgery and Trauma, Memorial Antalya Hospital, Zafer Mah. Yildirim Beyazit Cad. No. 91, Kepez, Antalya, Turkey

## Abstract

A 32-year-old male patient presented to our clinic with chronic left knee pain that was ongoing for about 1.5 years. The patient visited several times our clinic and the other clinics; conservative treatment (including rest, knee brace, and ice application with NSAIDs) was recommended by various different doctors. The anamnesis, physical examination, and plain radiography were nonspecific. Early MRI findings mislead us to believe it is bone marrow edema. One and half years with noneffective treatment, the knee pain persisted. At the latest visit intra-articular osteoid osteoma was suspected and the knee MRI with CT was employed. Even though the diagnosis of intra-articular osteoid osteoma often presents a challenge for the surgeons, with a present awareness of intra-articular osteoid osteomas which lack the characteristic sclerotic lesions and nidus on plain X-rays and the aid of multislice CT, a correct diagnosis which warrants proper treatment can be achieved. The possibility of osteoid osteomas, especially in young adults with persistent knee pain with unknown reasons that show normal plain radiographs results, must not be overlooked. The treatment method of these lesions should be customized depending on the location of the lesion, experience of the surgeon, and cost of method.

## 1. Introduction

Osteoid osteoma, first described by Jaffe in 1935 [1], is a common bone tumor that represents about 10% of all benign bone lesions [2]. According to Rizzoli Institute records (one of the famous institutions across the world regarding the bone tumors, the institution reported the data that has been collected between 1900 and 2009), it constitutes 18% of all benign tumors [3]. It is mainly seen within long bones and has a very typical appearance on X-rays with a tiny radiolucent zone, surrounded by reactive circumferential sclerosis (nidus) [4–6]. One of the characteristic clinical manifestations of this tumor is nocturnal pain, which can be alleviated using NSAIDs [7].

Approximately 13% of osteoid osteomas arise within the joint (intra-articular). Hip joint is the most commonly affected area, followed by the ankle, elbow, wrist, and knee [2].

Intra-articular location of the osteoid osteoma obscures classical symptoms and radiologic appearance, which, thus, can cause misdiagnosis and delayed proper treatment for the condition [8].

In this report, we would like to make a contribution to the literature by sharing our experience on a case with a lesion located on anteromedial tibia plateau (within joint capsule) and a delayed diagnosis by 1.5 years.

## 2. Case Report

**Figure 1:**
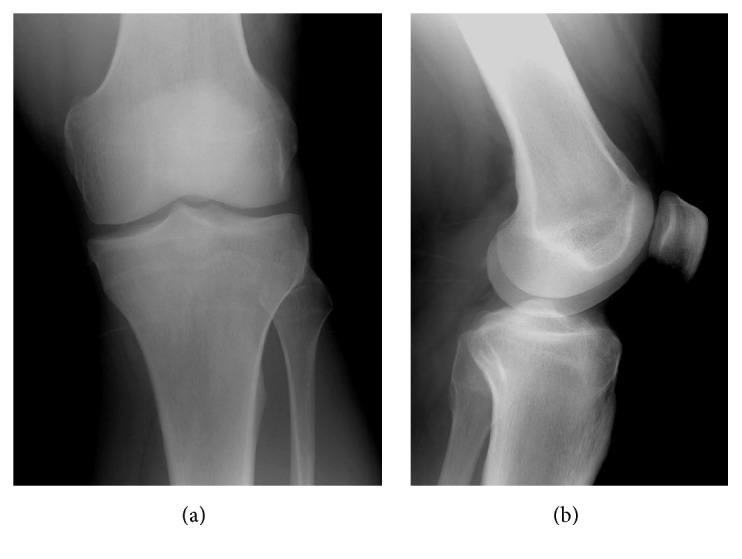
Normal findings at plain radiography.

A 32-year-old male patient presented to our clinic with chronic left knee pain that lasted for about 1.5 years. The patient visited several times our clinic before conservative treatment (including rest, knee brace, and ice application with NSAIDs) was recommended by two different doctors. The pain persisted during resting period and got worse during the night. NSAIDs caused temporary and partial pain relief. No significant medical stories from the patient or his family were seen. Physical examination showed some swelling and tenderness on anteromedial side of the left knee. Meniscal tests were negative, while the left quadriceps muscle showed moderate atrophy with an intact range of motion. Left knee radiographs showed no abnormalities (Figure 1).

**Figure 2:**
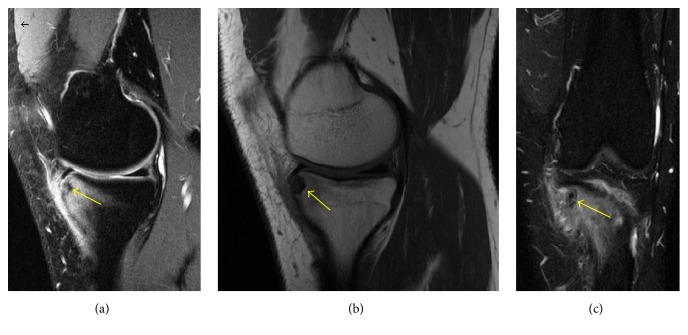
T2 weighted images (a and c) showed the hypointense lesion (yellow arrow) located at tibia anteromedial plateau with ill-defined bone marrow edema; (b) T1 weighted image showed the well-defined bordered hypointense lesion (yellow arrow).

When patient's MRI results were assessed, initial MRI of the patient, taken 1.5 years ago, at the beginning of the complaints, showed bone marrow edema. Second MRI, which is recent, showed a nidus-like view with bone marrow edema (Figure 2).

**Figure 3:**
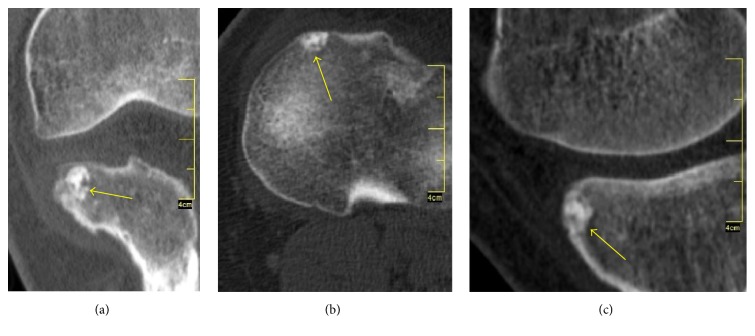
Coronal (a), axial (b), and sagittal (c) CT images showed the perinidal sclerosis with centrally calcified nidus (yellow arrow) consistent with an osteoid osteoma.

CT scans showed a small radiolucent zone, surrounded by reactive circumferential sclerosis, which is typical for osteoid osteoma (Figure 3). Based on physical examination findings and radiological results, the patient was diagnosed with intra-articular osteoid osteoma, arising from anteromedial tibia plateau. A surgical intervention for the open excision of the lesion was planned.

**Figure 4:**
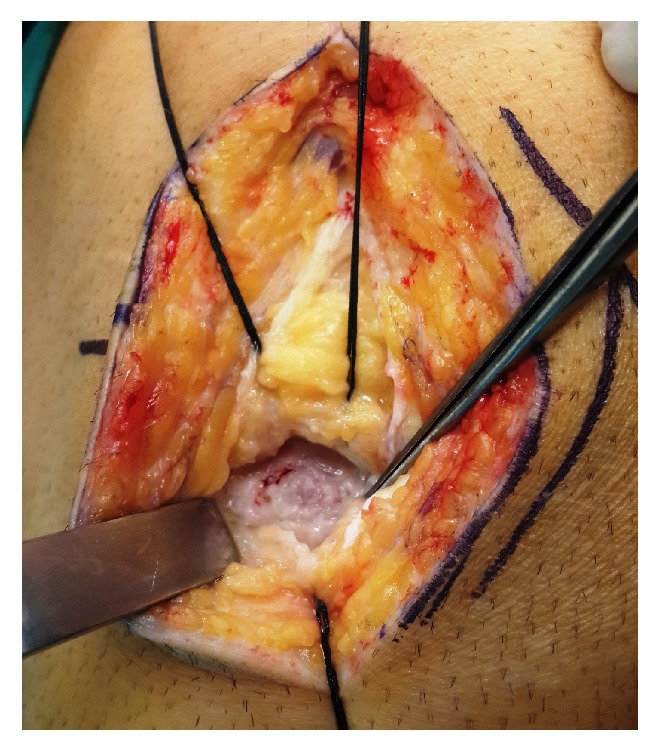
Intraoperative photo that shows surface discoloration over the lesion.

Under general anesthesia, pneumatic tourniquet was applied (350 mmHg) and a medial parapatellar incision was made. After opening the joint capsule, the Hoffa were stripped in the direction of lesion (towards medial), exposing the undersurface of anterior horn of medial meniscus. Intervention area was detected after the discolored and irregular lesion surface was marked (Figure 4). Tumor tissues were excised using gouges and curettes. Following the excision, the gap caused by the excision was filled with bone wax. Submeniscal tissues and capsule were closed using 2/0 Vicryl sutures. Following deflation of the pneumatic tourniquet, subcutaneous and cutaneous layers were sutured using appropriate materials.

**Figure 5:**
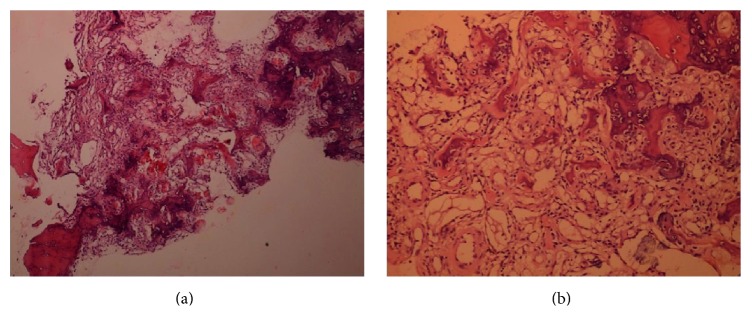
Photomicrograph of the lesion revealing abundant osteoid formation characteristic of an osteoid osteoma [H&E, ×4 obj. (a), ×10 obj. (b)].

Histopathological examination of the surgically removed lesion confirmed the initial diagnosis of osteoid osteoma (Figure 5). The patient was completely pain-free following the surgery and the joint gained full range of motion and the patient reported complete recovery from the condition, confirmed by follow-up visits on the 6th and 12th month of the surgery.

## 3. Discussion

In this article, we would like to highlight the difficulties on the diagnosis of the intra-articular osteoid osteoma. This atypical presentation with uncharacteristic radiological findings and the lack of trauma history usually directs the surgeon not to order unnecessary MRI/CT images, which causes delay on correct diagnosis.

Szendroi et al.'s [9] study compared the diagnosis periods for intra-articular osteoid osteoma and other locations. They reported that the mean diagnosis period was 22.6 months in intra-articular osteoid osteoma and 8.5 months for other locations.

Another study done by Kattapuram et al. [7] reported 3 main factors that contributed to this diagnostic challenge. First of those factors is the rarity of osteoid osteoma as a joint pain reason, as opposed to other causes. Second factor is the different manifestations of osteoid osteoma, such as joint effusion and/or response to salicylates [10–15], which can also be seen in other joint conditions which make the differential diagnosis more difficult. Third and last factor is that osteoid osteoma can cause complaints, even long before the lesion becomes apparent on radiological studies [11, 12, 16, 17]. Those three factors all contribute to the delay in diagnosis [13, 17, 18].

In addition to other authors, the low capability of producing new thick bone of the intracapsular periosteum was first underlined by Freiberger et al. [19]. Bauer et al.'s [20] case review that included 20 patients from 1972 to 1989 reported the importance of histopathological changes of the joint, affected by synovium-adjacent osteoid osteomas. They argued that the osteoid osteoma has the potential to cause lymphofollicular synovitis. In the following studies, other authors showed the arachidonic and metabolic pathways and production of prostaglandins (PGs) from the nidus, which caused synovitis in the first place [21–26].

These atypical anatomical, pathological, and physiological features of the affected intracapsular bone and adjacent synovium are all contributing factors in diagnosis delay of intra-articular osteoid osteoma. The patient can be subjected to redundant treatments such as unnecessary arthroscopies and/or arthrotomies with a delay in correct diagnosis and proper treatment [27].

As plain radiographs usually show no abnormalities in the joint, they have little value on the diagnosis. However, they are still required for elimination of other possible conditions and should be the first in line for diagnostic investigation in a normal diagnostic algorithm.

Another valuable investigation technique for classic extra-articular located osteoid osteomas is scintigraphy which shows the characteristic double density sign [28]. However, if the lesion is intra-articular, this sign is usually absent and lesion activity is limited within the joint due to associated synovitis, osteoporosis, and hyperemia [2]. Bone scintigraphy can be used to eliminate other possible lesions located on proximal femur that is associated with knee pain [29, 30].

MRI images can also be misleading for the surgeon, as the detection of synovitis or bone marrow edema during the early stages of the disease might lead the surgeon to misdiagnose the condition as arthritis or stress fracture of the bone. As in our case, nidus appearance usually develops at the late stages of the disease and is not evident on MRI until that time. Krause et al.'s [31] case report emphasized the stage-dependent MRI appearance by detailing the delay in nidus formation.

As opposed to our case, in the cases where the patient had a trauma or bruising history, the surgeons usually tend to suspect other causes such as meniscal tears, chondral lesions, and collateral ligament tears or bone bruising. In those situations, the surgeon is likely to order X-rays and MRI for diagnosis. Bone marrow edema view during early stages of the osteoid osteoma might lead the surgeon to misdiagnose the condition as a bone bruise or a stress fracture, therefore delaying the correct diagnosis, as in our case.

CT remains the method of choice for investigating intra-articular osteoid osteomas [2]. As in other bone lesions, CT is the main examination tool used for determining the lesion (nidus) and preoperative planning of the intervention. CT scans proved it to be a valuable tool for revealing small nidus, especially if those lesions are in the bones with complex anatomy such as spine or hip [32]. Pikoulas et al. [33] underlined the importance of slice thickness taken for the suspected nidus and recommended a thickness of at least 1 mm.

Recently, the new imagining methods such as SPECT/CT have gained popularity. SPECT/CT can be very useful especially in those cases when the osteoid osteoma is intra-articular and the X-ray and MRI are not informative. Sharma et al. [34] evaluated the data of 31 patients who had undergone ^99m^Tc–methylene diphosphonate (MDP) bone scintigraphy (BS) with SPECT/CT for clinically and/or radiologically suspected osteoid osteoma, retrospectively. In this study they compared the sensitivity, specificity, and accuracy of SPECT/CT, CT alone, and bone scintigraphy. They concluded that ^99m^Tc–MDP BS with SPECT/CT shows excellent diagnostic accuracy for osteoid osteoma and can be used as a one-stop imaging modality for the same. They also concluded that ^99m^Tc–MDP BS with SPECT/CT is superior to planar BS and CT alone for the diagnosis of suspected osteoid osteoma [34].

Latter Squier et al. [35] used 99mTc-methylene diphosphonate single-photon emission computed tomography/computed tomography for the imaging of suspected osteoid osteoma in the cervical spine. They demonstrated focal increased radiopharmaceutical activity in the right C2 lamina, which was associated with an osteolytic lesion with a central irregular sclerotic nidus. Surgical pathology confirmed an osteoid osteoma. They emphasized the value of this diagnostic tool on imaging osteoid osteoma which was located in cervical lamina.

Farid et al. [36] published an article in Clinical Nuclear Medicine Journal in which they emphasized the fusion of SPECT and CT could improve the specificity of abnormal scintigraphic findings. Unfortunately, in our hospital till to now we do not have this diagnostic tool. So we could not use this diagnostic advantage in our case.

Even though there were several reports on intra-articular osteoid osteomas in the distal femur [33], patella [37], and lateral tibia plateau [38, 39], case reports on anteromedial tibia plateau located lesions are quite few. Our literature scan revealed that only one study done by Harun et al. [40] reported a case of intra-articular osteoid osteoma of anteromedial tibia plateau that was treated by open surgery. Lesion location and surgery techniques used were like our case.

Despite the fact that CT-guided thermoablation is getting more and more popular for treating osteoid osteoma, it has several prerequisites such as assistance from a radiologist and the fact that the procedure has to be performed in either a CT room or an operating theatre equipped with a CT scanner. RF ablation might also cause cartilage degeneration when used for resecting subchondral osteoid osteomas [41, 42]. Therefore, we preferred open excision technique, as the lesion was located on the anteromedial edge of the tibia plateau that was inaccessible by arthroscopy.

In conclusion, here we presented an osteoid osteoma case that arises within knee joint which was successfully treated by open excision. Even though the diagnosis of intra-articular osteoid osteoma often presents a challenge for the surgeons, with a present awareness of intra-articular osteoid osteomas which lack the characteristic sclerotic lesions and nidus on plain radiography and the aid of multislice CT, a correct diagnosis which warrants proper treatment can be achieved. The possibility of osteoid osteomas, especially in young adults with persistent knee pain with unknown reasons that show normal plain radiograph results, must not be overlooked. The treatment method of these lesions should be customized depending on the location of the lesion, experience of the surgeon, and cost of method.

## Abbreviations

CT: Computed tomography
IAOO: Intra-articular osteoid osteoma
RF: Radiofrequency
MRI: Magnetic Resonance
NSAID: Nonsteroidal anti-inflammatory drugs
PGs: Prostaglandin(s).
